# Risk Factors for Delayed Cerebral Ischemia in Good‐Grade Patients With Aneurysmal Subarachnoid Hemorrhage

**DOI:** 10.1161/JAHA.122.027453

**Published:** 2022-12-06

**Authors:** Ilari Rautalin, Seppo Juvela, Michael L. Martini, Robert Loch Macdonald, Miikka Korja

**Affiliations:** ^1^ Department of Neurosurgery University of Helsinki and Helsinki University Hospital Helsinki Finland; ^2^ Icahn School of Medicine at Mount Sinai New York NY USA; ^3^ Community Health Partners Fresno CA USA

**Keywords:** aneurysmal subarachnoid hemorrhage, body mass index, delayed cerebral ischemia, good‐grade patients, hypertension, obesity, Cerebrovascular Disease/Stroke, Cerebral Aneurysm, Risk Factors, Ischemia, Lifestyle

## Abstract

**Background:**

A subset of good‐grade patients with aneurysmal subarachnoid hemorrhage (aSAH) develop delayed cerebral ischemia (DCI) that may cause permanent disabilities after aSAH. However, little is known about the risk factors of DCI among this specific patient group.

**Methods and Results:**

We obtained a multinational cohort of good‐grade (Glasgow Coma Scale 13–15 on admission) patients with aSAH by pooling patient data from 4 clinical trials and 2 prospective cohort studies. We collected baseline data on lifestyle‐related factors and the clinical characteristics of aSAHs. By calculating fully adjusted risk estimates for DCI and DCI‐related poor outcome, we identified the most high‐risk patient groups. The pooled study cohort included 1918 good‐grade patients with aSAH (median age, 51 years; 64% women), of whom 21% and 7% experienced DCI and DCI‐related poor outcome, respectively. Among men, patients with obesity and (body mass index ≥30 kg/m^2^) thick aSAH experienced most commonly DCI (33%) and DCI‐related poor outcome (20%), whereas none of the normotensive or young (aged <50 years) men with low body mass index (body mass index <22.5 kg/m^2^) had DCI‐related poor outcome. In women, the highest prevalence of DCI (28%) and DCI‐related poor outcome (13%) was found in patients with preadmission hypertension and thick aSAH. Conversely, the lowest rates (11% and 2%, respectively) were observed in normotensive women with a thin aSAH.

**Conclusions:**

Increasing age, thick aSAH, obesity, and preadmission hypertension are risk factors for DCI in good‐grade patients with aSAH. These findings may help clinicians to consider which good‐grade patients with aSAH should be monitored carefully in the intensive care unit.

**Clinical Perspective**

**What Is New?**

- In the study of 1918 good‐grade patients with aneurysmal subarachnoid hemorrhage, increasing age, thick amount of subarachnoid blood, preadmission hypertension, and obesity in men constituted the greatest risk factors for delayed cerebral ischemia and poor outcome related to it.
- According to these risk factors, the risk differences between patient subgroups were >6‐fold in women and >20‐fold in men.

**What Are the Clinical Implications?**

- Our findings may help clinicians to consider which of the patients with aneurysmal subarachnoid hemorrhage with good initial condition should be monitored carefully in the intensive care unit and which ones could be mobilized early to bed wards with a minimum risk of delayed neurological worsening.

**Nonstandard Abbreviations and Acronyms**
aSAH: aneurysmal subarachnoid hemorrhage
DCI: delayed cerebral ischemia

Although poor initial condition represents the most important predictor for unfavorable outcome after aneurysmal subarachnoid hemorrhage (aSAH), there is also a subset of initially good‐grade patients with aSAH who deteriorate and develop permanent deficits during hospitalization. A common cause of delayed deterioration is delayed cerebral ischemia (DCI).^1^, ^2^ Because DCI often acts as an underlying reason for poor outcome among good‐grade patients with aSAH who generally have the highest likelihood of full recovery, prevention of DCI among this patient group could be an effective strategy for increasing the number of patients with aSAH with favorable outcome.

Many patients with aSAH with otherwise good clinical condition are often monitored carefully in the intensive care unit (ICU) to prevent permanent ischemic deficits.^3^ To avoid unnecessarily prolonged ICU periods, prophylactic and therapeutic DCI treatments should be targeted for those at the highest risk. Therefore, it might be of value to identify good‐grade patients with aSAH with the lowest and highest DCI risk. However, apart from the amount of subarachnoid blood, little is known about the risk factors for DCI among good‐grade patients with aSAH.^4^ Therefore, our aim was to identify preadmission risk factors for DCI and DCI‐related poor outcome (ie, permanent disabilities), with a particular emphasis on the clinical characteristics that are easily defined and assessed on admission (eg, smoking, preadmission hypertension, and obesity). We hypothesized that by pooling data of 6 prospectively collected multinational study cohorts of good‐grade patients with aSAH, new risk factors for DCI and DCI‐related poor outcome in this patient group could be identified.

## METHODS

### Ethical Consideration

Local institutional review boards and ethical committees approved all included study cohorts, and informed consent was collected from all study participants. In addition, all included studies followed the ethical principles of the Declaration of Helsinki. The clinical trials that were conducted during the 21st century (CONSCIOUS‐1 [Clazosentan to Overcome Neurological Ischemia and Infarction Occurring after Subarachnoid Hemorrhage Trial], IHAST [The Intraoperative Hypothermia for Aneurysm Surgery Trial], and NEWTON‐1 [Nimodipine Microparticle to Enhance Recovery While Reducing Toxicity After Subarachnoid Hemorrhage Trial]) were registered to the public trials registry ClinicalTrials.gov. Detailed statistical analysis plan and pseudonymized data can be shared for qualified investigators providing a reasonable request to corresponding author.

### Study Cohort

Details of each study,^5^, ^6^, ^7^, ^8^, ^9^, ^10^ as well as the comparison of the main characteristics of the included cohorts,^11^ have been described previously. Briefly, we included the data of 4 clinical trials^6^, ^7^, ^8^, ^10^ and 2 prospective cohort studies,^5^, ^9^ which together included patients with aSAH from 14 countries and 93 health care units between 1985 and 2016. We only included patients with aSAH who were in good clinical grade (Glasgow Coma Scale 13–15 or World Federation of Neurological Surgeons grades I–III) on admission.

### Baseline Data Collection

We collected the data pertaining to patients with aSAH: age, sex, height, weight, clinical grade on admission (Glasgow Coma Scale and/or World Federation of Neurological Surgeons), smoking history (no versus yes), presence of preadmission hypertension (no versus yes), amount of subarachnoid blood (no/thin versus thick [Fisher grade 3 or modified Fisher grade 3–4]), as well as the location and treatment modality (neurosurgical versus endovascular treatment) of the ruptured aneurysm (please see detailed definitions by cohorts in Table S1). We used body mass index (BMI) as the obesity variable. We investigated the effect of obesity both by continuous assessment (per each SD increase of BMI) and in weight extremes by categorizing patients with aSAH according to the World Health Organization's obesity classifications, with slight modifications: (1) low BMI (BMI <22.5 kg/m^2^; the combination of underweight and the lower half of normal weight categories), (2) moderate BMI (BMI 22.5–29.9 kg/m^2^; the combination of overweight and the higher half of normal weight categories), and (3) high BMI (BMI ≥30 kg/m^2^; the combination of all obese categories). These slight modifications (dichotomization of the normal weight category and combining the obese categories) were done to preserve a sufficient sample size in low and high BMI categories, even for sex‐specific subgroup analyses.

### Outcome Assessment

In line with the general recommendations,^12^ we used clinically observed DCI and DCI‐related poor outcome as the main outcome variables. The clinical definition of DCI was consistent in all 6 studies: gradual development of focal neurological deficit or deterioration in the level of consciousness after ruling out other causes (eg, infections and electrolyte disorders) (Table S1). We also assessed the number of patients with DCI‐related poor outcome to identify the permanent deficits after aSAH. The DCI‐related poor outcome was defined as patients who experienced DCI in hospital and had subsequently poor outcome (Glasgow Outcome Scale 1–3^13^) at 3 months after aSAH.

### Statistical Analysis

We used an unconditional logistic regression model to calculate risk estimates (odds ratios [ORs] and 95% CIs) for DCI and DCI‐related poor outcome. In addition, we used partially adjusted (for age, sex, and study cohort) and fully adjusted multivariable calculations to estimate the independent effects of baseline variables. Fully adjusted models included age, sex, and study cohort, as well as the variables that had a significant association (*P*<0.05) with risk of DCI or DCI‐related poor outcome in the partially adjusted models. Along with pooled estimates, we also calculated age‐ and sex‐adjusted risk estimates for each study cohort, and evaluated the between‐cohort heterogeneity of the significant cohort‐wide risk factors using the *I* ^2^ test. Moreover, we evaluated whether the risk factors for outcomes differed by sex, and used the likelihood ratio test to calculate *P* values for multiplicative interactions (effect modifications) caused by sex. Finally, we determined the lowest and highest risk groups for DCI and DCI‐related poor outcome by calculating the proportion of patients who experienced either outcome in the subgroups of observed independent risk factors. Because one of the clinical trials (IHAST^10^) excluded all severely obese (BMI ≥35 kg/m^2^) patients with aSAH, we excluded the patients of that cohort from the BMI‐related analysis to avoid the distorting effect of a likely selection bias. To minimize the risk of type I error (false‐positive findings), we limited the overall number of univariate analyses by focusing only on the routinely collected risk factor variables at baseline. Moreover, the conclusions were based on the adjusted multivariable models, not on single unadjusted univariate analyses. Stata version 16.1 (Stata Corp, College Station, TX) was used for all statistical analyses.

## RESULTS

### General Characteristics

Table 1 presents the baseline characteristics of the included 1918 good‐grade patients with aSAH. The median age was 51 years, and 64% of the patients were women. Most (88%) patients underwent surgical treatment. Of the 1918 good‐grade patients, 408 (21%) experienced DCI, and 129 (7%) had DCI‐related poor outcome at 3 months.

**Table 1:** Demographic Data by Sex

Variable	Men	Women	Overall
No. of patients (% of all)			
Overall	686 (100.0)	1232 (100.0)	1918 (100.0)
Juvela cohort	94 (13.7)	90 (7.3)	184 (9.6)
Enoxaparin trial	66 (9.6)	69 (5.6)	135 (7.0)
CONSCIOUS‐1	95 (13.9)	224 (18.2)	319 (16.6)
NEWTON‐1	14 (2.0)	17 (1.4)	31 (1.6)
IHAST	345 (50.3)	655 (53.2)	1000 (52.1)
SHOP	72 (10.5)	177 (14.4)	249 (13.0)
Age, median (IQR), y	49.0 (40.0–57.0)	51.6 (43.0–61.0)	51.0 (42.0–59.0)
Preadmission hypertension, n (%)			
No	454 (66.2)	726 (58.9)	1180 (61.5)
Yes	219 (31.9)	481 (39.0)	700 (36.5)
Missing	13 (1.9)	25 (2.0)	38 (2.0)
Smoking, n (%)			
No	251 (36.6)	604 (49.0)	855 (44.6)
Yes	426 (62.1)	612 (49.7)	1038 (54.1)
Missing	9 (1.3)	16 (1.3)	25 (1.3)
BMI, median (IQR), kg/m^2^	25.9 (23.4–29.0)	24.7 (22.1–28.6)	25.0 (22.6–28.8)
BMI categories, n (%)			
Low BMI (BMI <22.5 kg/m^2^)	53 (7.7)	164 (13.3)	217 (11.3)
Moderate BMI (BMI 22.5–29.9 kg/m^2^)	215 (31.3)	282 (22.9)	497 (25.9)
High BMI (BMI ≥30 kg/m^2^)	63 (9.2)	114 (9.3)	177 (9.2)
Missing*	355 (51.8)	672 (54.6)	1027 (53.6)
Amount of subarachnoid blood, n (%)			
No/thin	322 (46.9)	554 (45.0)	876 (45.7)
Thick	359 (52.3)	672 (54.6)	1031 (53.8)
Missing	5 (0.7)	6 (0.5)	11 (0.6)
Aneurysm location, n (%)			
ICA	130 (19.0)	435 (35.3)	565 (29.5)
ACA/ACoA	245 (35.7)	327 (26.5)	572 (29.8)
MCA	254 (37.0)	315 (25.6)	569 (29.7)
Posterior circulation	56 (8.2)	150 (12.2)	206 (10.7)
Missing	1 (0.2)	5 (0.4)	6 (0.3)
Treatment modality			
Neurosurgical clipping	614 (89.5)	1078 (87.5)	1692 (88.2)
Endovascular coiling	72 (10.5)	154 (12.5)	226 (11.8)

ACA indicates anterior cerebral artery; ACoA, anterior communicating artery; BMI, body mass index; CONSCIOUS‐1, Clazosentan to Overcome Neurological Ischemia and Infrarction Occurring after Subarachnoid Hemorrhage Trial; ICA, internal carotid artery; IHAST, the Intraoperative Hypothermia for Aneurysm Surgery Trial; IQR, interquartile range; MCA, middle cerebral artery; NEWTON‐1, Nimodipine Microparticles to Enhance Recovery While Reducing Toxicity After Subarachnoid Hemorrhage Trial and SHOP, The Columbia University Subarachnoid Hemorrhage Outcomes Project.

* Patients from IHAST trial were excluded from the BMI‐related analysis because the trial excluded all patients with aneurysmal subarachnoid hemorrhage with BMI ≥35 kg/m^2^.

### Risk Factors for DCI and DCI‐Related Poor Outcome

According to the partially adjusted analysis (adjusted for age, sex, and study cohort), we found that increasing age, preadmission hypertension, and thick aSAH were associated with an increased risk of DCI and DCI‐related poor outcome, whereas low BMI was associated with decreased risk estimates (Table 2). In addition, patients with a ruptured aneurysm in the anterior communicating artery or anterior cerebral artery more often experienced DCI. In the fully adjusted models (adjusted for age, sex, study cohort, and variables with significant associations in the partially adjusted model), the associations remained similar (Table 2). When the study population was stratified by sex, the only significant risk factor difference was observed for the effect of BMI on DCI‐related poor outcome (*P*=0.024 for interaction between BMI and sex in the risk of DCI‐related poor outcome). In fact, each SD (=5.6‐unit) increase of BMI was associated with a >2‐fold (OR, 2.43 [95% CI, 1.30–4.56]) increase in the risk of DCI‐related poor outcome in men, whereas no such association was observed in women (OR, 0.94 [95% CI, 0.70–1.26]). Similarly but slightly insignificantly obese men (BMI ≥30 kg/m^2^) had an almost 3‐fold (OR, 2.67 [95% CI, 0.95–7.49]) increased risk of DCI‐related poor outcome compared with men with moderate BMI (BMI 22.5–29.9 kg/m^2^). The impact of thick aSAH and increasing age as risk factors for DCI and DCI‐related poor outcome differed moderately by study cohorts (*I* ^2^=22.5%–51.7%). In comparison, no significant between‐cohort heterogeneity was found for the associations of preadmission hypertension and obesity with the risk of DCI or DCI‐related poor outcome (*I* ^2^=0.0%) (Table S2).

**Table 2:** Partially and Fully Adjusted ORs With 95% CIs for DCI and DCI‐Related Poor Outcome by Baseline Variables

Variable	ORs (95% CIs) for DCI		ORs (95% CIs) for DCI‐related poor outcome	
	Partially adjusted model	Fully adjusted model	Partially adjusted model	Fully adjusted model
Age				
Per each SD increase	1.14 (1.01–1.28)*	1.05 (0.93–1.19)	1.66 (1.36–2.01)*	1.45 (1.17–1.79)*
Sex				
Men	Reference	Reference	Reference	Reference
Women	1.07 (0.84–1.35)	1.10 (0.86–1.41)	1.18 (0.79–1.76)	1.20 (0.80–1.80)
Hypertension				
No	Reference	Reference	Reference	Reference
Yes	1.36 (1.07–1.74)*	1.36 (1.06–1.74)*	2.01 (1.35–2.98)*	2.01 (1.35–3.00)*
Smoking				
No	Reference	NA	Reference	NA
Yes	1.21 (0.96–1.54)		1.08 (0.73–1.59)	
BMI				
Per each SD increase	1.09 (0.93–1.29)	NA	1.10 (0.88–1.39)	NA
Low BMI (BMI <22.5 kg/m^2^)	0.59 (0.39–0.91)*	0.67 (0.42–1.07)	0.48 (0.24–0.93)*	0.50 (0.25–1.02)
Moderate BMI (BMI 22.5–29.9 kg/m^2^)	Reference	Reference	Reference	Reference
High BMI (BMI ≥30.0 kg/m^2^)	0.82 (0.53–1.26)	0.80 (0.50–1.28)	0.85 (0.46–1.55)	0.90 (0.48–1.69)
Amount of subarachnoid blood				
No/thin	Reference	Reference	Reference	Reference
Thick	2.27 (1.78–2.88)*	2.23 (1.75–2.85)*	2.55 (1.67–3.91)*	2.65 (1.72–4.09)*
Aneurysm location				
ICA	Reference	Reference	Reference	NA
ACA/ACoA	1.49 (1.11–2.00)*	1.49 (1.10–2.01)*	1.18 (0.72–1.93)	
MCA	1.02 (0.75–1.38)	0.99 (0.72–1.35)	1.13 (0.68–1.87)	
Posterior circulation	0.74 (0.47–1.16)	0.77 (0.48–1.21)	1.33 (0.70–2.51)	
Treatment modality				
Neurosurgical clipping	Reference	NA	Reference	NA
Endovascular coiling	0.92 (0.57–1.49)		1.32 (0.68–2.56)	

ACA indicates anterior cerebral artery; ACoA, anterior communicating artery; BMI, body mass index; DCI, delayed cerebral ischemia; ICA, internal carotid artery; MCA, middle cerebral artery; NA, not applicable; and OR, odds ratio.

* *P*<0.05.

### High‐ and Low‐Risk Patient Groups for DCI and DCI‐Related Poor Outcome

**Figure 1:**
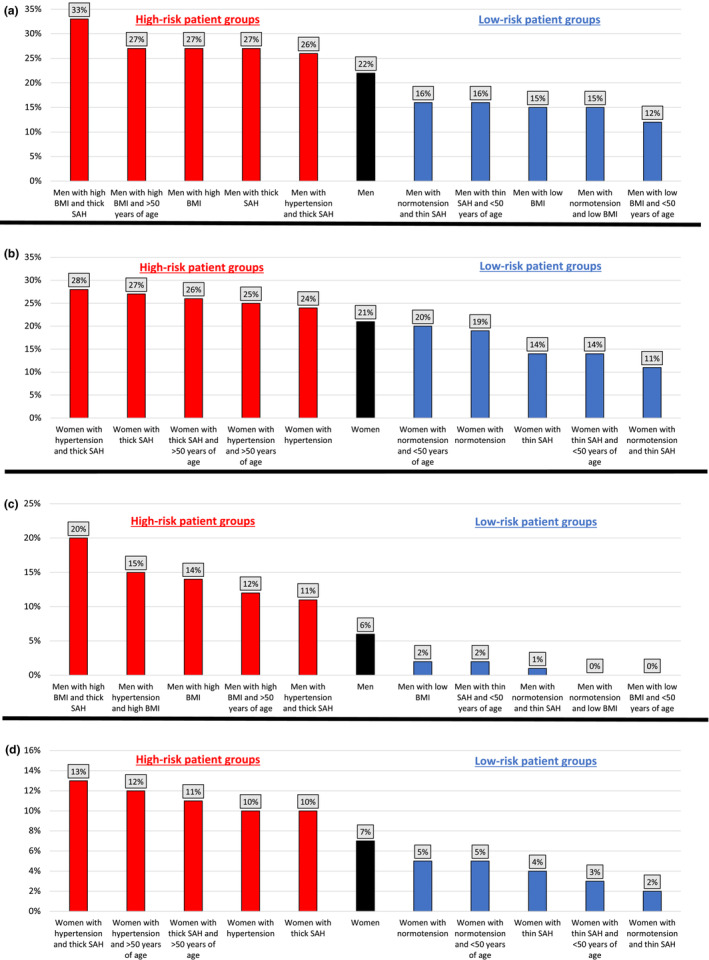
Five lowest‐ and highest‐risk patient groups for delayed cerebral ischemia (DCI) and DCI‐related poor outcome after aneurysmal subarachnoid hemorrhage (SAH). **A**, Proportion of good‐grade men who experienced DCI. **B**, Proportion of good‐grade women who experienced DCI. **C**, Proportion of good‐grade men who experienced DCI‐related poor outcome. **D**, Proportion of good‐grade women who experienced DCI‐related poor outcome. BMI indicates body mass index.

On the basis of the significant risk factors in the fully adjusted analyses, we identified the good‐grade patient populations with aSAH with the lowest and highest risks of DCI and DCI‐related poor outcome (Figure 1 and Tables S3 and S4). Among men, those with a high BMI (BMI ≥30 kg/m^2^) and thick aSAH most often experienced DCI (33%) and DCI‐related poor outcome (20%). Conversely, normotensive or young (aged <50 years) men with a low BMI (BMI <22.5 kg/m^2^) experienced more infrequently DCI‐related poor outcome. Among women, the highest rates of DCI (28%) and DCI‐related poor outcome (13%) were observed in those with preadmission hypertension and thick aSAH, whereas the lowest rates were found in normotensive women with thin aSAH (11% and 2%, respectively) (Figure 1).

## DISCUSSION

In addition to thick aSAH, which is the well‐established risk factor for DCI,^4^ our analysis revealed that increasing age, preadmission hypertension, and obesity in men were associated with the increased risk of DCI and DCI‐related poor outcome among patients with aSAH with good clinical condition on admission. Although the risk factor findings were similar for both outcomes, the risk differences were especially notable for permanent deficits. We found that women with preadmission hypertension and thick aSAH had a 6‐fold higher risk of DCI‐related poor outcome compared with normotensive women with no/thin aSAH. Similarly, the risk of DCI‐related poor outcome was >20 times higher in men with a high BMI and thick aSAH compared with normotensive or young men with a low BMI. If other studies will confirm our results, we believe that these absolute risk differences could help clinicians to identify the good‐grade patients with aSAH who could benefit from early mobilization with a minimal risk of delayed worsening and ICU readmission. On the other hand, careful subacute‐phase monitoring in the ICU may be reasonable for the groups of patients identified as high risk, despite their favorable baseline clinical condition. Nevertheless, because of observational design, no treatment recommendations can be made entirely on these findings. Moreover, the benefits and drawbacks of a prolonged immobilization of elderly and obese good‐grade patients with aSAH should be further studied.

**Figure 2:**
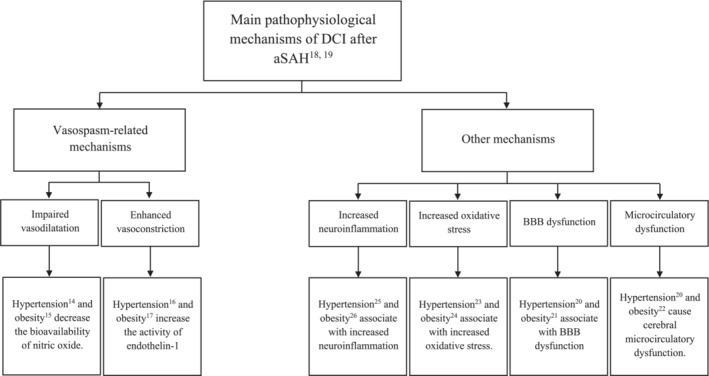
Theoretical pathophysiological explanations for the adverse effect of preadmission hypertension and obesity on delayed cerebral ischemia (DCI) after aneurysmal subarachnoid hemorrhage (aSAH). BBB indicates blood‐brain barrier.

Our results are supported by previous pathophysiological findings, as several in vivo studies have related both obesity and hypertension^14^, ^15^, ^16^, ^17^ to underlying causes of aSAH‐related cerebral vasospasm^18^, ^19^ (Figure 2). Moreover, previous studies have found that people with obesity and hypertension frequently have blood‐brain barrier dysfunction,^20^, ^21^ cerebral microcirculatory dysfunction,^20^, ^22^ an increased level of oxidative stress,^23^, ^24^ and systemic neuroinflammation,^25^, ^26^ all of which have been suggested to play a major role in DCI development^18^, ^19^ (Figure 2). It is also possible that the adverse effects of preadmission hypertension and high BMI on patient outcomes may relate to more severe bleeding. On the other hand, because we observed poor outcome even among patients with high BMI and/or preadmission hypertension but without thick aSAH, preadmission hypertension and obesity do not necessarily lead to a severe aSAH and automatically to DCI and poor outcome. Why obesity seems to be more hazardous in men than in women remains to be studied in the future.

A previous study,^11^ which did not assess the effect of obesity on DCI and DCI‐related poor outcome, found that an increasing BMI was associated with an increased risk of poor outcome after aSAH, especially among men. Although several other studies have associated obesity with an increased risk of DCI after aSAH^27^, ^28^, ^29^, ^30^ in all grades of patients (not focusing on good‐grade patients), negative^31^, ^32^, ^33^, ^34^, ^35^, ^36^ and controversial^37^ findings have also been reported. Some of these controversial findings have been related to the obesity paradox (a controversial theory suggesting that patients with obesity may have a better prognosis than normal weight patients after aSAH),^38^ but several other reasons, such as wide heterogeneity of study populations (which consisted of varying proportions of good‐ and poor‐grade patients with aSAH), retrospective and single‐center data collections, inaccurate obesity measurements, small sample sizes, missing information for possible confounders, and lack of sex‐stratified analyses, more likely explain previous conflicting results. In the current study, we minimized the risk of many shortcomings by including a large sample of multinational patients with aSAH with similar baseline condition, consistent data collection, consistent obesity measurements, uniform definition of DCI, and prospective identification of most potential confounders at the time of aSAH. Therefore, we believe that this study may provide reliable estimates about the relationship of obesity and DCI, at least among good‐grade patients with aSAH. In terms of hypertension, previous studies (none focusing on good‐grade patients with aSAH) have associated preadmission hypertension with an increased risk of sudden‐death aSAH,^39^ worse survival,^40^ worse functional outcome,^41^ and ischemic complications after aSAH.^4^, ^29^, ^41^

Our study also has limitations. First, because the data set did not contain information about the treatment delay or the time interval from bleeding to the observed DCI, it is possible that some of the deficits may have occurred immediately after surgery or later than 14 days after aSAH, and can thus be attributed to reasons other than DCI. However, given that all other explanations for neurological deterioration had to be excluded in each individual cohort, and we only included patients with aSAH with good clinical condition on admission (instances and causes of deterioration may be more objectively defined among good‐grade patients), we believe that the impact of a few possibly misidentified DCIs on the present findings is minimal. Second, the data set did not allow us to discern whether the observed DCIs were temporary or if they caused permanent deficits. Therefore, we aimed to elaborate permanent impairments by determining the patients with aSAH who experienced DCI in hospital and poor outcome 3 months after aSAH. Although it is possible that the poor outcome of some patients with aSAH may be attributed to causes other than DCI (eg, to treatment complications, rebleeding, hydrocephalus, thromboembolic complications, or postoperative infections), we believe that the proportion of such patients is small because of the strict definitions of DCI and our inclusion of only good‐grade patients with aSAH, as discussed above. Moreover, because these other possible causes would rather dilute than strengthen the effect size of observed risk factors, we believe that their effect on our conclusions would be negligible. Third, it is also possible that some of the patients with aSAH who experienced moderate disabilities (Glasgow Outcome Scale 4) at 3 months may have had permanent DCI‐related deficits that were not identified. On the other hand, as the main risk factor, findings remained similar even when DCI‐related poor outcome was defined as patients with in‐hospital DCI and Glasgow Outcome Scale 1 to 4 at 3 months (results not shown), and the Glasgow Outcome Scale cutoff point for poor outcome did not have a substantial impact on our results. Nevertheless, future studies should assess the permanent DCI‐related deficits with more accurate outcome measures. Fourth, because most included patients with aSAH underwent surgical treatment, the findings are not perhaps applicable to endovascularly treated patients. In addition, not only operative treatment modalities but also general neurocritical care has evolved substantially during the study period (from 1985 to 2016), and this has likely caused some treatment effect changes. On the other hand, as our results did not differ significantly by treatment modality, between the first (1985–2000) and the second half (2000–2016) of the study period (results not shown) or between each included study cohort from different decades (Table S2), we believe that the time‐related effects on the results are not substantial. Moreover, robust advances, particularly in the DCI treatment, that would also lead to an improved outcome have remained limited during the past decades.^19^ Fifth, 4 of the included studies allocated their patients with aSAH by different treatment interventions (enoxaparin trial by administration of enoxaparin, CONSCIOUS‐1 by administration of clazosentan, NEWTON‐1 by administration of intraventricular nimodipine, and IHATS by mild intraoperative hypothermia), and this has further increased the treatment variation within the pooled study cohort. However, as only one of the studies (NEWTON‐1; representing only 2% of our study cohort) reported a significant effect of the used intervention on the occurrence of DCI and poor outcome, and because we found only modest heterogeneity between the risk factor results of each study cohort (Table S2), these treatment differences unlikely distort our findings. Sixth, because we did not have data on smoking habits and actual blood pressure values, our results may underestimate the adverse effects of smoking and preadmission hypertension. This may also partly explain why smoking, an important risk factor for aSAH death^39^, ^40^ and DCI in all grades of patients,^4^ had only a modest trend toward an increased risk of DCI in our study. Because heavy smokers frequently have poor clinical condition on admission and a relatively high likelihood of death from aSAH before hospitalization,^39^ most heavy smokers may have been excluded from this good‐grade study cohort,^40^ and therefore the effect size to study smoking effects on DCI and DCI‐related poor outcome in good‐grade patients was still insufficient. In addition, 65% of patients aged <50 years were classified as smokers at baseline, whereas 45% of patients aged ≥50 years were smokers. Because younger patients with aSAH tend to have more favorable outcome than older ones, this may further confound analyses of smoking effects on DCI and DCI‐related outcome. Seventh, in addition to smoking habits and blood pressure values, data of various other previously reported preadmission (eg, comorbidities, such as diabetes), on‐admission (eg, increased systemic inflammation markers and neuroimaging risk scores), and postadmission (eg, hydrocephalus and other immediate aSAH‐related complications) risk factors for DCI^4^ were only collected in a few studies and most commonly with different methods and at different time points. Therefore, we decided to exclude such variables from further analyses. On the other hand, because most of these previous risk factor findings are based on single, small, and retrospective studies that focus on all‐grade patients with aSAH,^4^ we believe that missing data will not invalidate the reported main conclusions. Nevertheless, future studies with more comprehensive and prospective data collection are needed. Finally, because our biggest study cohort (the IHAST trial^10^; representing approximately half of the included patients) excluded all severely obese (BMI ≥35 kg/m^2^) patients with aSAH, we excluded patients from IHAST from the BMI‐related analyses. Although this exclusion limited the sample size of our BMI‐related analyses, it allowed for less biased estimates of the effect of obesity on adverse outcomes following aSAH. In fact, if we had included the patients from IHAST in these analyses, the findings would have remained similar, with diluted effect sizes. For example, the risk increase for DCI‐related poor outcome would have been ≈2‐fold (fully adjusted OR, 1.93 [95% CI, 0.90–4.13]) among men with obesity compared with men with moderate BMI.

## CONCLUSIONS

An increasing age, thick aSAH, preadmission hypertension, and obesity in men may be risk factors for DCI and DCI‐related poor outcome in good‐grade patients with aSAH. If confirmed, our findings may be of importance when considering whether good‐grade patients with aSAH should be monitored carefully in the ICU or mobilized early to regular bed wards.

## Sources of Funding

Dr Rautalin received personal research grants from Juho Vainio Foundation, Alfred Kordelin Foundation, Finnish Medical Foundation, and Aarne Koskelo Foundation. In addition, open access was funded by Helsinki University Library. The funders had no role in the design and conduct of the study; in collection, management, analysis, and interpretation of the data; or in preparation, review, or approval of the manuscript.

## Disclosures

None.

## Supporting information

Tables S1–S4
