# Supplementary material for: Risk Factors for Delayed Cerebral Ischemia in Good‐Grade Patients With Aneurysmal Subarachnoid Hemorrhage
Source: J Am Heart Assoc. 2022 Dec 6;11(23):e027453. doi: 10.1161/JAHA.122.027453 (PMC9851459; doi:10.1161/JAHA.122.027453)
Supplement: Supplementary file 1 — Tables S1–S4 [file JAH3-11-e027453-s001.pdf]

SUPPLEMENTAL MATERIAL

Table S1. Definitions of baseline characteristics and outcomes by study cohort.

Variable	Juvela cohort	Enoxaparin trial	CONSCIOUS-1 trial	NEWTON-1 trial	IHAST trial	SHOP cohort
Baseline characteristics						
Pre-admission hypertension	Antihypertensive medication or high blood pressure values (over 160/95) measured repeatedly before aSAH	Antihypertensive medication or high blood pressure values (over 160/95) measured repeatedly before aSAH	Diagnosed hypertension before aSAH	Diagnosed hypertension before aSAH	Diagnosed hypertension or using antihypertensive medication before aSAH	Diagnosed hypertension before aSAH
Smoking	Current smoker at the time of aSAH	Current smoker at the time of aSAH	Current smoker at the time of aSAH	Current smoker at the time of aSAH	Current smoker (or quitted ≤ 6 months ago) at the time of aSAH	Current smoker and smoked > 100 cigarettes at the time of aSAH
Thick aSAH	Fisher grade 3 (> 1mm thick layer in vertical layers of CT scan)	Fisher grade 3 (> 1mm thick layer in vertical layers of CT scan)	Modified Fisher grade 3–4 (> 4mm thick diffuse or local layer)	Modified Fisher grade 3–4 (> 4mm thick diffuse or local layer)	Fisher grade 3 (> 1mm layer in vertical layers or localized subarachnoid clot)	Fisher grade 3 (Thick aSAH clot)
Outcome variables						
DCI	Gradual development of focal neurological deficit or a deterioration in the level of consciousness due to unknown reason. Both temporary and permanent deficits were included.	Gradual development of focal neurological deficit or a deterioration in the level of consciousness due to unknown reason. Both temporary and permanent deficits were included.	Gradual development of focal neurological deficit or a deterioration in the level of consciousness due to unknown reason. Not apparent immediately after aneurysm occlusion. Both temporary and permanent deficits were included.	Gradual development of focal neurological deficit or a deterioration in the level of consciousness due to unknown reason. Occurred within 14 days after aSAH. Both temporary and permanent deficits were included.	Gradual development of focal neurological deficit or a deterioration in the level of consciousness due to unknown reason. Occurred within 14 days after aSAH. Both temporary and permanent deficits were included.	Gradual development of focal neurological deficit or a deterioration in the level of consciousness due to unknown reason. Occurred at least 48 hours after operation. Both temporary and permanent deficits were included.
DCI-related poor outcome	DCI in hospital and poor outcome (Glasgow Outcome Scale I-III) at three months after aSAH	DCI in hospital and poor outcome (Glasgow Outcome Scale I-III) at three months after aSAH	DCI in hospital and poor outcome (Glasgow Outcome Scale I-III) at three months after aSAH	DCI in hospital and poor outcome (Glasgow Outcome Scale I-III) at three months after aSAH	DCI in hospital and poor outcome (Glasgow Outcome Scale I-III) at three months after aSAH	DCI in hospital and poor outcome (Glasgow Outcome Scale I-III) at three months after aSAH

aSAH = aneurysmal subarachnoid hemorrhage; CT = computed tomography; DCI = delayed cerebral ischemia

Table S2. Between-cohort heterogeneity between the observed risk differences of delayed cerebral ischemia (DCI) and DCI-related poor outcome.

Risk factor	I^2-test for between-cohort heterogeneity	
	DCI	DCI-related poor outcome
Increasing age	37.1%	0.0%
Thick aSAH	22.5%	51.7%
Pre-admission hypertension	0.0%	0.0%
Obesity in men	0.0%	0.0%

aSAH = aneurysmal subarachnoid hemorrhage; DCI = delayed cerebral ischemia

Table S3. Proportion of male aneurysmal subarachnoid hemorrhage (aSAH) patients who suffered from delayed cerebral ischemia (DCI) or DCI-related poor outcome by observed fully adjusted risk factors.

	DCI, % (n all)	DCI-related poor outcome, % (n of all)
Men	21.5 (684)	6.1 (686)
Low risk patients		
Men, age under 50 years	21.9 (365)	4.4 (367)
Men, normotension	21.2 (452)	4.6 (454)
Men, low BMI	15.1 (53)	1.9 (53)
Men, thin aSAH	15.9 (321)	3.1 (322)
Men, age under 50 years, normotension	19.7 (290)	3.8 (292)
Men, age under 50 years, low BMI	11.8 (34)	0.0 (34)
Men, age under 50 years, thin aSAH	15.5 (181)	1.7 (182)
Men, normotension, low BMI	14.9 (47)	0.0 (47)
Men, normotension, thin aSAH	15.6 (225)	1.3 (226)
Men, low BMI, thin aSAH	21.9 (32)	3.1 (32)
High risk patients		
Men, age over 50 years	21.0 (319)	8.2 (319)
Men, hypertension	21.9 (219)	9.1 (219)
Men, high BMI	27.0 (63)	14.3 (63)
Men, thick aSAH	26.8 (358)	8.9 (359)
Men, age over 50 years, hypertension	17.8 (152)	9.9 (152)
Men, age over 50 years, high BMI	27.3 (33)	12.1 (33)
Men, age over 50 years, thick aSAH	24.9 (177)	10.7 (177)
Men, hypertension, high BMI	22.2 (27)	14.8 (27)
Men, hypertension, thick aSAH	26.4 (125)	11.2 (125)
Men, high BMI, thick aSAH	32.5 (40)	20.0 (40)

aSAH = aneurysmal subarachnoid hemorrhage; BMI = body mass index; DCI = delayed cerebral ischemia

Table S4. Proportion of female aneurysmal subarachnoid hemorrhage (aSAH) patients who suffered from delayed cerebral ischemia (DCI) or DCI-related poor outcome by observed fully adjusted risk factors.

	DCI % (n all)	DCI-related poor outcome % (n all)
Women	21.2 (1232)	7.1 (1 232)
Low-risk patients		
Women, age under 50 years	20.6 (579)	5.2 (579)
Women, normotension	19.2 (726)	5.2 (726)
Women, thin aSAH	14.3 (554)	3.8 (554)
Women, age under 50 years, normotension	19.5 (400)	4.5 (400)
Women, age under 50 years, thin aSAH	13.5 (303)	2.6 (303)
Women, normotension, thin aSAH	11.0 (335)	2.1 (335)
High-risk patients		
Women, age over 50 years	21.8 (653)	8.7 (653)
Women, hypertension	24.3 (481)	10.0 (481)
Women, thick aSAH	26.9 (672)	9.8 (672)
Women, age over 50 years, hypertension	25.1 (319)	11.6 (319)
Women, age over 50 years, thick aSAH	26.2 (397)	11.1 (397)
Women, hypertension, thick aSAH	28.2 (266)	12.8 (266)

aSAH = aneurysmal subarachnoid hemorrhage; DCI = delayed cerebral ischemia
